# Phosphatidylinositol 3-kinase pathway activation in breast cancer brain metastases

**DOI:** 10.1186/bcr3071

**Published:** 2011-12-01

**Authors:** Barbara Adamo, Allison M Deal, Emily Burrows, Joseph Geradts, Erika Hamilton, Kimberly L Blackwell, Chad Livasy, Karen Fritchie, Aleix Prat, J Chuck Harrell, Matthew G Ewend, Lisa A Carey, C Ryan Miller, Carey K Anders

**Affiliations:** 1 Department of Medicine, Division of Hematology-Oncology, CB 7305, University of North Carolina, Chapel Hill, NC 27599, USA; 2 Department of Human Pathology, Integrated Therapies in Oncology Unit, University of Messina, Messina 98125, Italy; 3 Lineberger Comprehensive Cancer Center, 170 Manning Drive, CB 7350, University of North Carolina, Chapel Hill, NC 27599, USA; 4 Department of Biostatistics and Clinical Data Management, Lineberger Comprehensive Cancer Center, Manning Drive, CB 7350, University of North Carolina, Chapel Hill, NC 27599, USA; 5 Department of Pathology, Duke University Medical Center, Box 3712, Durham, NC 27710, USA; 6 Department of Medicine, Division of Hematology-Oncology, 382 Hanes Building, Duke University Medical Center, Box 102382, Durham, NC 27710, USA; 7 Department of Pathology, Carolinas Medical Center, P.O. Box 32187, Charlotte, NC 28232, USA; 8 Department of Pathology, Mayo Clinic, 13400 East Shea Boulevard, Rochester, MN 85259, USA; 9 Department of Genetics, 120 Mason Farm Road, CB#7264, University of North Carolina, Chapel Hill, NC 27599, USA; 10 Department of Pathology & Laboratory Medicine, CB#7525, University of North Carolina, Chapel Hill, NC 27599, USA; 11 Department of Neurosurgery, CB 7250, University of North Carolina, Chapel Hill, NC 27599, USA

## Abstract

**Introduction:**

Activation status of the phosphatidylinositol 3-kinase (PI3K) pathway in breast cancer brain metastases (BCBMs) is largely unknown. We examined expression of phospho(p)-AKT, p-S6, and phosphatase and tensin homologue (PTEN) in BCBMs and their implications for overall survival (OS) and survival after BCBMs. Secondary analyses included PI3K pathway activation status and associations with time to distant recurrence (TTDR) and time to BCBMs. Similar analyses were also conducted among the subset of patients with triple-negative BCBMs.

**Methods:**

p-AKT, p-S6, and PTEN expression was assessed with immunohistochemistry in 52 BCBMs and 12 matched primary BCs. Subtypes were defined as hormone receptor (HR)+/HER2-, HER2+, and triple-negative (TNBC). Survival analyses were performed by using a Cox model, and survival curves were estimated with the Kaplan-Meier method.

**Results:**

Expression of p-AKT and p-S6 and lack of PTEN (PTEN-) was observed in 75%, 69%, and 25% of BCBMs. Concordance between primary BCs and matched BCBMs was 67% for p-AKT, 58% for p-S6, and 83% for PTEN. PTEN- was more common in TNBC compared with HR+/HER2- and HER2+. Expression of p-AKT, p-S6, and PTEN- was not associated with OS or survival after BCBMs (all, *P *> 0.06). Interestingly, among all patients, PTEN- correlated with shorter time to distant and brain recurrence. Among patients with TNBC, PTEN- in BCBMs was associated with poorer overall survival.

**Conclusions:**

The PI3K pathway is active in most BCBMs regardless of subtype. Inhibition of this pathway represents a promising therapeutic strategy for patients with BCBMs, a group of patients with poor prognosis and limited systemic therapeutic options. Although expression of the PI3K pathway did not correlate with OS and survival after BCBM, PTEN- association with time to recurrence and OS (among patients with TNBC) is worthy of further study.

## Introduction

The incidence of brain metastases (BMs) is approximately 15% among women newly diagnosed with metastatic breast cancer (BC) [1]. This figure likely underestimates the true incidence, as autopsy studies report a 30% incidence of BMs among women with advanced disease [2]. Current therapeutic interventions include corticosteroids, whole-brain radiotherapy, neurosurgical resection, stereotactic radiosurgery, and systemic chemotherapy [3]. Despite these treatment strategies, prognosis among patients with BCBMs remains poor, with a median overall survival of approximately 6 months [4,5]. Although targeted agents show promise in the treatment of advanced extracranial BC, challenges in delivery of these agents to the central nervous system (CNS) include properties inherent to the blood barrier (that is, efflux mechanisms) and our incomplete understanding the biology underlying BCBMs. Moreover, optimal therapeutic targets within BCBM are largely unknown.

Previous studies indicate that the phosphatidylinositol 3-kinase (PI3K) pathway plays a critical role in the initiation and progression of human BC, and alterations in this pathway have been identified in approximately 50% of these tumors [6,7]. PI3K pathway activation occurs in response to extracellular signals via either growth-factor receptor or integrin pathways. On its recruitment to the cellular membrane via receptor-mediated activation, the p110α catalytic subunit of PI3K phosphorylates phosphatidylinositol-4,5-bisphosphate (PIP2) at the 3' position of the inositol ring, generating PIP3 [8]. PIP3 recruits phospholipid-binding domain containing proteins, particularly AKT, to the plasma membrane. Phosphorylated (p-)AKT, the primary downstream effector of PI3K signaling, moves from the cytoplasm to the nucleus to initiate its downstream effects. This cascade, including activation of the mammalian target of rapamycin (mTOR) and its downstream effectors, p70S6 kinase and 4E-binding protein-1, affects a number of cellular processes, including proliferation and motility, which clinically translate into endocrine and chemotherapy resistance and worse cancer-specific survival [6,9-12]. The PI3K/AKT pathway is negatively regulated by PTEN (phosphatase and tensin homologue), a lipid phosphatase that removes the 3-phosphate from PI(3,4)P2 and PI(3,4,5)P3, thus inactivating the signaling cascade [13]. Therefore, loss of PTEN contributes to the activation of the PI3K/AKT signaling cascade through inhibition of degradation of both PI(3,4)P2 and PI(3,4,5)P3.

To date, alterations and activation of the PI3K/AKT pathway are well established in the initiation and progression of extracranial human BC [6,8-10,14,15]. However, the contribution of this important signaling pathway to the pathogenesis of BCBMs has yet to be fully elucidated. This is of clinical importance as small-molecule inhibitors of the PI3K/AKT/mTOR pathway are in development and show promising activity in the treatment of primary brain tumors, suggesting sufficient blood-brain barrier penetration to elicit therapeutic effects [16,17]. In this study, we quantitated the expression of the PI3K pathway biomarkers p-AKT, p-S6, and PTEN, and evaluated the prognostic implications, primarily overall survival (OS) and survival after BCBMs, of PI3K activation status in BCBMs. As secondary, exploratory end points, we evaluated the associations between PI3K pathway activation and time to distant recurrence (TTDR) and time to BCBM. Finally, similar analyses were also conducted among the subset of patients with triple-negative BCBM.

## Materials and methods

### Patients

BCBMs (*n *= 52), including a subset with matched primary BCs (*n *= 12), from 52 patients treated at the University of North Carolina at Chapel Hill (UNC) (52%) and Duke University (48%) between 1991 and 2008, were studied. Clinical data, including age, race, stage of primary BC at diagnosis (as per the 2003 Sixth Edition of the American Joint Committee on Cancer [AJCC]), treatment history (including local and systemic therapies in the adjuvant and metastatic settings), recurrence, and vital status were available for 50 patients. Given the retrospective nature of clinical data collection, complete information was not available for all 50 patients; thus, denominators may vary throughout the article. This study was approved, and waivers of consent were granted by Institutional Review Boards at both UNC and Duke.

### Immunohistochemistry

Immunohistochemistry (IHC) was performed on 5-μm formalin-fixed, paraffin-embedded tissue sections on coated glass slides by using a Dako Autostainer (DakoCytomation, Carpinteria, CA). Monoclonal antibodies were applied for 30 to 60 minutes at room temperature (with the exception of HER2, which was incubated overnight at 4°C) and detected by using avidin-biotin chemistry (Vectastain Elite Kit 6102; Vector, Burlingame, CA) and diaminobenzidine (Innovex NB314SB; Innovex Biosciences, Richmond, CA) as chromogen. Signal contrast was maximized by counterstaining with hematoxylin (DakoCytomation Mayer hematoxylin S3309), rinsing in deionized water, and placement in a bluing solution (Richard-Allan Scientific 7301; Richard-Allan Scientific, Kalamazoo, MI). The following primary antibodies and dilutions were used: p-AKT (Ser473, 1:10, Cell Signaling, Danvers, MA); PTEN clone 6H2.1 (1:25; Cascade Biosciences, Winchester, MA); p-S6 ribosomal protein (1:50, Ser235/236; Cell Signaling); ER clone 1D5 (1:50; Dako), PR clone 16 (1:70; Vision BioSystems, Norwell, MA); and HER2 clone CB11 (1:100; BioGenex, San Ramon, CA). For each antibody, primary breast tumor tissue was used as a positive control. Technical negative controls omitting the primary antibody using primary breast tumor tissue were also used. IHC was performed on all 52 cases for p-AKT, p-S6, and PTEN. IHC for ER, PR, and HER2 expression was additionally performed on 38 cases. Because of limited tissue, four cases were stained for HER2 only, and three cases were stained for ER and PR only.

### Immunohistochemistry scoring

IHC stains were scored by two surgical pathologists (KF and CL). Nuclear ER and PR staining were scored from 0 to 8 by using the Allred system [17], and Allred scores of 0 to 2 and 3 to 8 were defined as negative and positive, respectively. HER2 was scored by using the current American Society of Clinical Oncology ASCO)/College of American Pathologists (CAP) guidelines [18]. Membranous immunoreactivity was scored (0 and 1+ indicates negative; 2+, indeterminate; and 3+, positive for overexpression), and the percentage of tumor cells staining positive was visually estimated.

For p-AKT, p-S6, and PTEN, an H score was calculated by multiplying the fraction of positively stained tumor (percentage) by staining intensity (0, 1+, 2+, or 3+). In subsequent statistical analyses, H scores were classified as negative (0 to 9), low (10 to 100), medium (101 to 200), or high (201 to 300) [18].

### Identification of breast cancer subtypes

BC subtype was defined by IHC receptor status of the BCBM as follows: Hormone receptor (ER and/or PR) HR+/HER2-, triple-negative (ER-/PR-/HER2-, TN), and HER2+. In five BCBM cases for which ER, PR, and HER2 IHC data were not available, receptor status of the BCBM was obtained per the clinical database and was used to assign subtype. In three of five cases in which HER2 re-staining was indeterminate (IHC = 2+), HER2 classification was based on available clinical data including either IHC and/or FISH (fluorescence *in situ *hybridization). In the other two cases, HER2 2+ was classified as negative for the purposes of this analysis.

### Gene-expression microarray analysis

Normalized gene-expression data from two publicly available datasets [19,20] were evaluated. In Harrell *et al*. [19] (GSE26338), we analyzed the expression of *PTEN *in a combined cohort of 855 primary BC patients that were followed, and the first site of distant recurrence was retrospectively annotated, including a subset of 42 patients in whom the first site of relapse (or one of the first sites) was the brain. The intrinsic molecular subtype calls were used as provided in Harrell *et al*. [19]. In Zhang *et al*. [20] (GSE14018), we evaluated the expression of *PTEN *across 36 unpaired BC brain, liver, bone, and lung metastasis samples. In both datasets, all *PTEN *probes were averaged into a single expression value.

### Statistical analysis

The Kaplan-Meier method and log-rank test were used to compare differences among survival curves, and Cox regression analysis was used to evaluate possible predictors in the time-to-event outcomes. Overall survival (OS) was defined as the time from diagnosis of primary BC to death or last contact. CNS-specific survival (CNS survival) was defined as the time from the date of BCBM to the date of death or last follow-up. Time to distant recurrence (TTDR) was defined as the time from primary BC diagnosis to date of distant recurrence. Time to CNS recurrence (TTCNS) was defined as the time from primary BC diagnosis to date of CNS metastases; for those patients whose initial distant recurrence included the CNS, this time was the same as the time to distant recurrence. Differences in *PTEN *gene expression across the various intrinsic molecular subtypes and between brain metastases and other distant metastastic sites was evaluated by using a Wilcoxon rank sum test. Associations with relapse-free survival were evaluated by using the log-rank test, and Cox regression; *PTEN *expression was categorized as low/medium or high based on combining the lower two tertiles. Statistical analyses were performed with SAS 9.2 statistical software (SAS Institute, Inc., Cary, NC) and R v.2.8.1 http://cran.r-project.org.

## Results

### Patient and tumor characteristics

The clinical characteristics of the study population are presented in Table 1. The median age at diagnosis of primary BC was 48 years (range, 26 to 72 years). Sixty-eight percent of patients were Caucasian, 30%, African-American, and 2%, other ethnicities. Fifty percent of patients were Stage II, and 29% were Stage III at the time of surgery for primary BC.

**Table 1:** Patient, tumor, and treatment characteristics

Characteristic	All patients
	*N *(%)
**No. of patients**	50
**Median age**	48 (26-72 years)
**< 40 years**	12 (24%)
**≥40 years**	38 (76%)
**Ethnicity**	
**Caucasian**	34 (68%)
**African American**	15 (30%)
**Other**	1 (2%)
**Stage**	*N *= 34
**I**	4 (12%)
**II**	17 (50%)
**III**	10 (29%)
**IV**	3 (9%)
**IHC subtype**	*N *= 43
**HR+/HER2-**	12 (28%)
**HR-/HER2- (TN)**	19 (44%)
**HER2+**	12 (28%)
**Systemic therapy for primary BC**	
**Chemotherapy**	44/48 (92%)
**Endocrine therapy (for HR+/HER2-)**	6/11 (55%)
**Trastuzumab (for HER2+)**	2/12 (17%)
**Systemic therapy for metastatic BC**	
**Chemotherapy**	28/38 (74%)
**Endocrine therapy (for HR+/HER2-)**	7/10 (70%)
**Her2-directed therapy (for HER2+)**	6/9 (67%)
**BCBM therapy**	*N *= 47
**Cranial XRT**	25 (53%)
**Radiosurgery**	4 (9%)

BC subtype was assigned based on IHC staining of BCBM for 43 patients, and subtype distribution was as follows: 28% HR+/HER2-, 44% TN, and 28% HER2+. Subtype concordance between primary BC and associated BCBM was 57% (four of seven). Of the three cases that were discordant, two HER2+ primary BC lacked HER2 staining in the matched BCBM, whereas one TN primary BC gained HR positivity (HR+/HER2-) in the matched BCBM.

### Overview of systemic and local therapies

Ninety-two percent of patients received systemic chemotherapy with curative intent (29% and 71% in the neoadjuvant and adjuvant settings, respectively) for their primary BC (Table 1), whereas 55% (HR+/HER2-) received endocrine therapy, and 17% (HER2+) received trastuzumab.

In the metastatic setting, 95% (36 of 38) of patients received some form of systemic therapy; with 32% receiving one line, and 63% receiving two or more lines of therapy. Seventeen percent (six of 35) received systemic therapy both before and after development of CNS metastases; 20% only before and 63% only after diagnosis of BCBM. Therapies in the metastatic setting included the following: chemotherapy (74%), endocrine therapy (70%, HR+ only), and HER2-directed therapy (67%, HER2+ only). Fifty-three percent received cranial radiation (XRT) for BCBM; 9% received radiosurgery. No difference in OS or CNS survival was seen between those who did or did not receive cranial XRT (*P *= 0.7, 0.5).

### Expression of PI3K pathway biomarkers in breast cancer brain metastases

**Figure 1:**
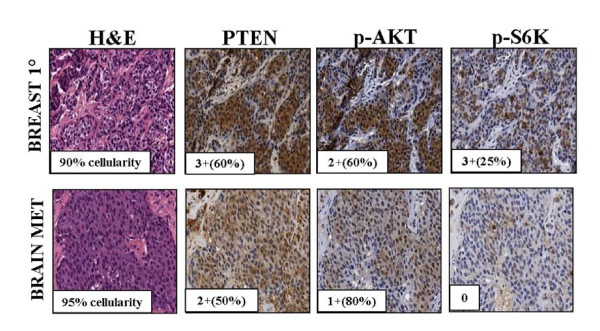
**Expression of p-AKT, pS6, and *PTEN *by immunohistochemistry in a primary breast tumor and its paired brain metastasis**.

Activation of the PI3K pathway in BCBM was determined by evaluating the expression of p-AKT, p-S6, and PTEN with IHC. Expression of p-AKT and p-S6 was positive (H score, 10 to 300) in 75% and 69% of BCBM, respectively (Table 2a and Figure 1). Twenty-five percent of BCBMs lacked PTEN expression (PTEN-, H score, 0 to 9).

**Table 2:** Expression of PI3K pathway biomarkers in breast cancer brain metastases

H score	PTEN		p-AKT		p-S6	
**Negative** **(0 to 9)**	13 (25%)		13 (25%)		16 (31%)	
**Low** **(10 to 100)**	19 (37%)		19 (37%)		25 (48%)	
**Medium** **(101 to 200)**	7 (13%)	39 (75%)	10 (19%)	39 (75%)	7 (13%)	36 (69%)
**High** **(201 to 300)**	13 (25%)		10 (19%)		4 (8%)	

No significant association was found between BCBM subtype and PI3K pathway status for p-AKT, p-S6, or PTEN (all *P *> 0.3). Interestingly, PTEN- was more frequent among the TN BCBM (seven of 19, 37%) compared with HR+/HER2- (two of 12, 17%) and HER2+ (three of 12, 25%) BC (*P *= 0.3). Concurrent PI3K pathway activation (p-AKT and/or p-S6 H score ≥10) and PTEN- (H score, 0 to 9) was present in 15% of 52 BCBMs. A larger proportion (five of 19, 26%) of BCBMs arising from patients with TNBC showed this IHC pattern, compared with 8% (one of 12) of the HR+/HER2- and 17% (two of 12) of the HER2+ patients (*P *= 0.5).

### Concordance of PI3K expression between brain metastases and primary breast tumors

PI3K pathway biomarkers status in primary BC and their matched BCBM was concordant in 67%, 58%, and 83% of 12 cases for p-AKT, p-S6, and PTEN, respectively (Table 3), and both gains and losses of which were evident for each biomarker evaluated.

**Table 3:** Concordance of PI3K biomarkers between primary breast tumors and matched breast cancer brain metastases (*n *= 12)

Biomarker	Gain	Loss	Concordance
PTEN	1 (8.3%)	1 (8.3%)	10 (83.3%)
p-AKT	2 (16.6%)	2 (16.6%)	8 (66.6%)
p-S6	3 (25.0%)	2 (16.6%)	7 (58.3%)

### Survival outcomes according to breast cancer subtype

**Figure 2:**
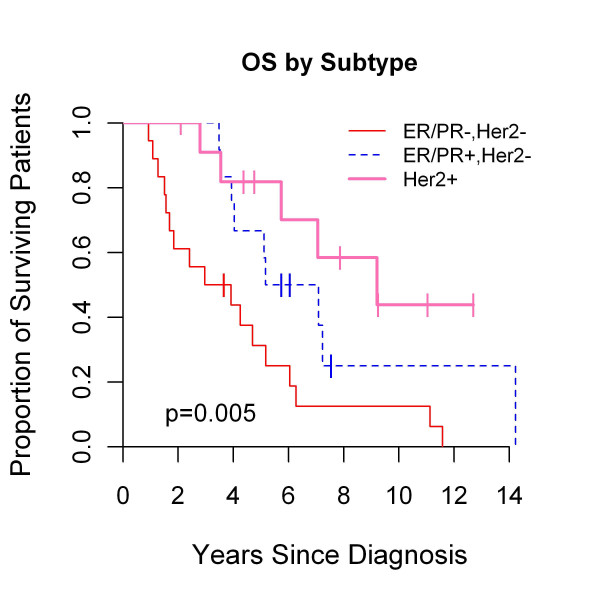
**Overall survival, defined as time from primary breast cancer diagnosis to death, by breast cancer subtype**.

**Figure 3:**
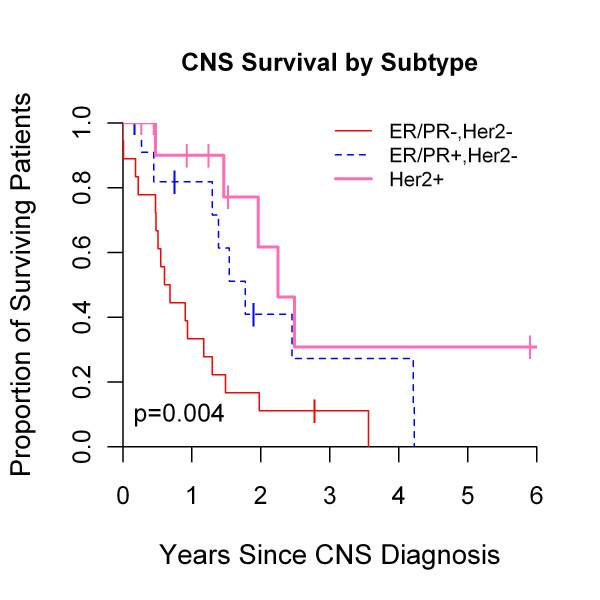
**Survival after breast cancer brain metastases as determined by breast cancer subtype**.

Prior reports suggested that BC prognosis is dependent on IHC subtype, as TN portends inferior outcome regardless of systemic therapy [21-24]. The prognostic implication of IHC subtype within BCBMs was examined. The median follow-up for survivors was 7 years, and 74% (37 of 50) of patients have died. As shown in Figure 2, median overall survival was 6.1 years, 3.4 years, and 9.2 years for HR+/HER2-, TN, and HER2+ subtypes, respectively (*P *= 0.005). Median survival after BCBM diagnosis was 1.8, 0.64, and 2.3 years for HR+/HER2-, TN, and HER2+, respectively (*P *= 0.004, Figure 3). Median time to distant recurrence was 3.7, 1.8, and 3.2 years for HR+/HER2-, TN, and HER2+, respectively (*P *= 0.11), and median time to CNS recurrence was 3.7, 1.9, and 3.8 years for HR+/HER2-, TN, and HER2+, respectively (*P *= 0.2, data not shown).

### Survival outcomes by expression of p-AKT, p-S6, and PTEN

**Figure 4:**
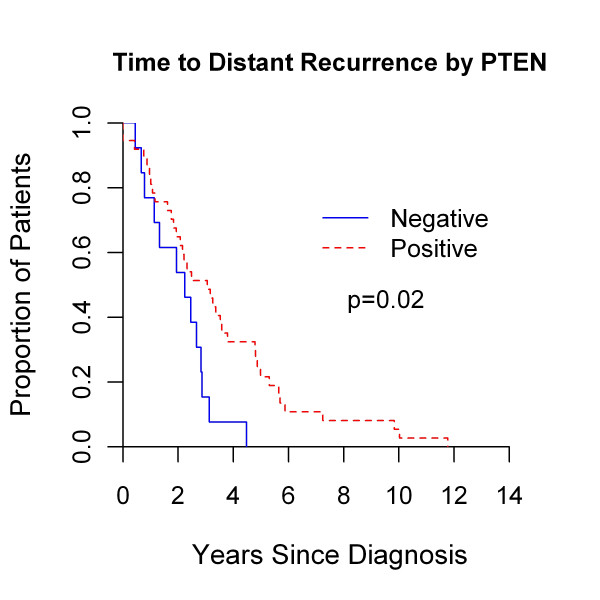
**Time to distant recurrence from primary breast cancer diagnosis by *PTEN *negativity versus positivity as determined by brain metastases immunohistochemistry**.

**Figure 5:**
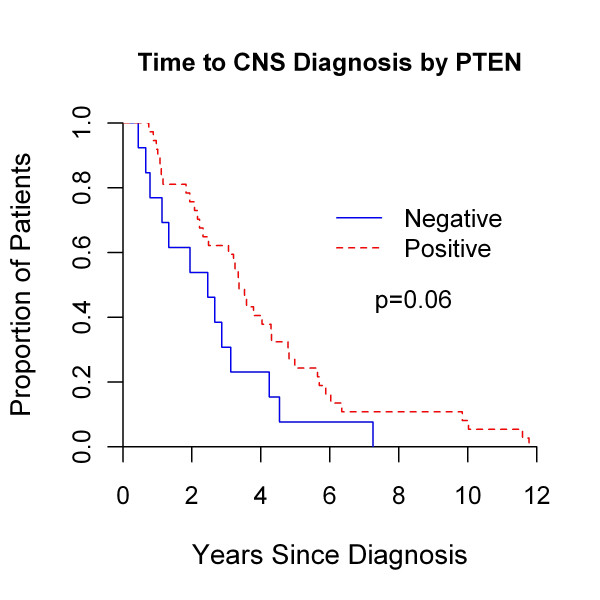
**Time to central nervous system recurrence from primary breast cancer diagnosis by *PTEN *negativity versus positivity, as determined by brain metastases immunohistochemistry**.

The prognostic implications of p-AKT, p-S6, and PTEN expression in BCBMs were evaluated. Expression of p-AKT, p-S6, and PTEN (H score, < 10 versus ≥10) was not associated with the primary outcome of overall survival or survival after BCBMs (*P *≥ 0.06 for all analyses; data not shown). In secondary analyses, neither expression of p-AKT nor p-S6 was associated with time to distant or CNS recurrence (*P *≥ 0.05 for all analyses, data not shown). Although not associated with an inferior overall survival from primary BC diagnosis (5.1 versus 5.7 years; *P *= 0.16) or survival after BCBM (2.0 versus 1.4 years, *P *= 0.9; data not shown), PTEN- BCBM was associated with shorter time to both distant (2.2 versus 3.1 years; *P *= 0.02; Figure 4) and CNS recurrence (2.5 versus 3.4 years; *P *= 0.06; Figure 5) even when stratified by TNBC (see later section) in exploratory analyses. Moreover, 5-year freedom from distant recurrence were 0 and 22% (95% confidence interval (CI), 10% to 36%) for PTEN- versus PTEN+ BCBMs. Similarly, 5-year freedom from CNS recurrence was 7.6% (95% CI, 0.5% to 29%) and 24% (95% CI, 12% to 39%) for these two groups, respectively.

To confirm that these results were not influenced by receipt of systemic therapy, we evaluated the proportion of patients who received treatment by PTEN status. No difference was found in receipt of systemic chemotherapy, either in the curative (10 of 12, 83% versus 34 of 36, 94%; *P *= 0.26) or advanced setting (eight of nine, 89%, versus 20 of 29, 69%; *P *= 0.4), between patients with either PTEN- or PTEN+ BCBM, respectively. Interestingly, a higher proportion of PTEN- BCBM patients (nine of 11, 82%) received cranial XRT for BCBM compared with those with PTEN+ BCBM (16 of 26, 44%; *P *= 0.04).

### Survival by PTEN status among patients with triple-negative breast cancer

**Figure 6:**
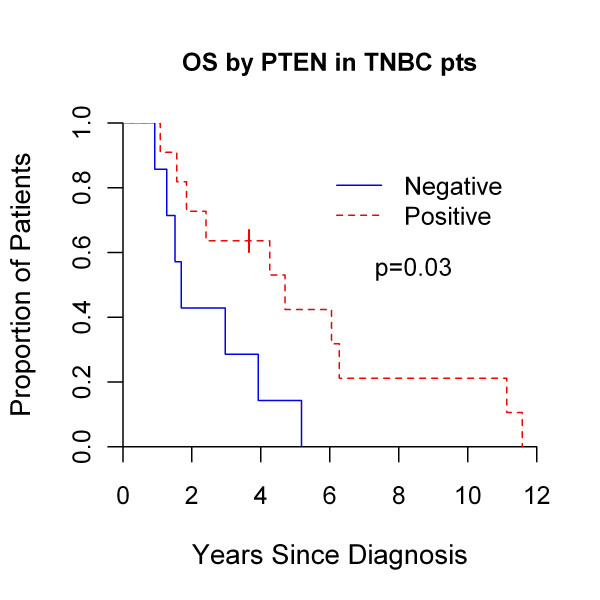
**Overall survival by *PTEN *status as determined by brain metastases immunohistochemistry in patients with triple-negative breast cancer**.

Recognizing the association between TNBC and PTEN expression, we evaluated the prognostic significance of PTEN expression within the TN BCBM subset as a secondary and exploratory outcome. PTEN- TN BCBMs were associated with inferior overall survival (1.7 years) compared with PTEN+ BCBM (4.7 years; *P *= 0.03; Figure 6). PTEN status had no significant effect on overall survival in patients with non-TN BCBM (PTEN-, 8.2 years; PTEN+, 7.1 years; *P *= 0.8, data not shown). No significant effect of PTEN status on time to distant recurrence, time to CNS recurrence, or survival after BCBM was noted for patients with either TN or non-TN BCBM (all *P *≥ 0.5; data not shown). However, time to distant recurrence (1.3 versus 1.9 years; *P *= 0.08) and time to CNS recurrence (1.3 versus 2.5; *P *= 0.11) was shorter for patients with PTEN-, TN BCBM.

### Impact of subtype and PTEN status on patient outcome

Consistent with the Kaplan-Meier analyses, the TN IHC subtype was found to be associated with worse overall survival, time to distant and CNS recurrence, and survival after BCBM in univariable Cox regression analyses (all, *P *≤ 0.06) (Table 4). PTEN- was associated with more rapid time to distant recurrence (hazard ratio (HR), 2.2; *P *= 0.025); a borderline association between PTEN- and shorter time to CNS recurrence was observed (HR, 1.8; *P *= 0.07).

**Table 4:** 

Univariable analyses									Multivariable analyses							
	OS		TTDR		TTCNS		OS CNS		OS		TTDR		TTCNS		OS CNS	
Variable	HR	*P*	HR	*P*	HR	*P*	HR	*P*	HR	*P*	HR	*P*	HR	*P*	HR	*P*
PTEN (H score, negative vs. positive)	1.7	0.16	2.2	0.025	1.8	0.07	0.95	0.9	1.9	0.1	2.4	0.02	2	0.06	0.93	0.86
Subtype (TN vs. non-TN)	3.1	0.003	1.9	0.04	1.8	0.06	3.2	0.003	3.2	0.002	2	0.04	1.8	0.06	3.2	0.003

On multivariable Cox regression analyses, the association between subtype and overall survival, time to distant recurrence, and survival after CNS metastases remained significant when controlling for PTEN status (all, *P *≤ 0.04). Similarly, the association between PTEN- and shorter time to distant recurrence (HR, 2.4; *P *= 0.02) and time to CNS recurrence (HR, 2; *P *= 0.06) remained when controlling for subtype among patients with BCBMs.

### Evaluation of *PTEN *gene expression across the intrinsic molecular subtypes and brain metastases

**Figure 7:**
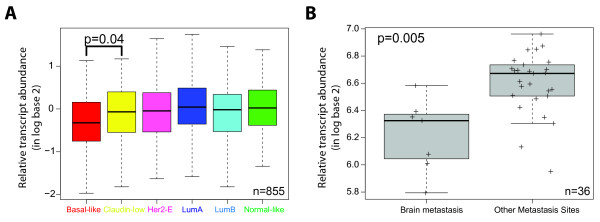
**Evaluation of *PTEN *gene expression in breast cancer samples from two publicly available data sets **[19,20]. **(a) ***PTEN *expression across the intrinsic molecular subtypes of 855 primary tumors of the combined cohort in Harrell *et al*. [19]. **(b) ***PTEN *expression in a panel of unpaired breast cancer brain metastases (*n *= 7) and other distant sites (*n *= 29). The colored boxes represent the interquartile range (IQR); the bar indicates the median value; whiskers show the 1.5 × IQR.

To further explore the association of PTEN- with triple-negative disease and brain metastases, we interrogated two publicly available gene-expression microarray data sets that included: (a) 855 primary breast cancers with annotated intrinsic subtype and relapse-free survival data [19], and (b) 36 unpaired brain, lung, liver, and bone BC metastases [20]. First, we evaluated the expression of the *PTEN *gene across the intrinsic molecular subtypes in the Harrell *et al*. [19] dataset. As shown in Figure 7a, basal-like tumors showed an overall lower expression of *PTEN *compared with the other subtypes of BC. Second, we observed that *PTEN *expression was expressed at lower levels in BCBMs compared with other distant metastatic sites (Figure 7b). Although we cannot rule out that this observation is due to the fact that these brain metastases were largely of the basal-like subtype, whereas bone and liver metastasis were more of the luminal and HER2-enriched subtypes, these data support the association of lower levels of *PTEN*, basal-like tumors, and the development of brain metastases.

### Survival outcomes based on *PTEN *gene expression

**Figure 8:**
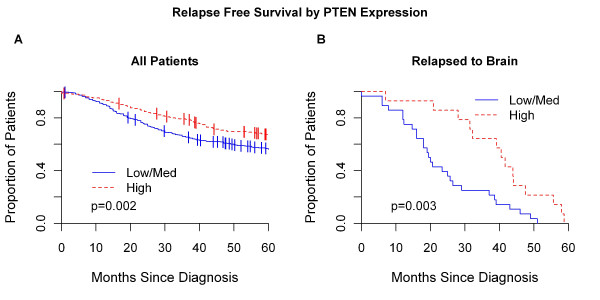
**Evaluation of *PTEN *gene expression in primary tumors with outcome at 5 years in Harrell *et al***. [19] combined data set. **(a) **Relapse-free survival in the entire data set (*n *= 855) based on *PTEN *expression. **(b) **Time to brain recurrence (as the first site of relapse) based on *PTEN *expression in the subset of patients that relapse to the brain in the first 5 years (*n *= 42). In both survival analyses, tumors in the highest *PTEN *expression tertile of the entire data set were identified as high *PTEN *expressers, and the rest, as low *PTEN *expressers. *P *values shown here have been adjusted for ER status and intrinsic molecular subtype.

Further to explore the association of *PTEN*- with poor outcome, we evaluated the Harrell *et al*. [19] combined microarray data set. In all patients (*n *= 855), lower levels of *PTEN *expression were found to be associated with poor prognosis at 5 years (*P *= 0.002), even when adjusted for ER status (*P *= 0.012) and ER status plus intrinsic molecular subtype (*P *= 0.025; Figure 8a). This suggests that *PTEN *is not just recapitulating the poor prognosis of the basal-like subtype, and supports our IHC-based findings that lack of *PTEN *expression is also found in the other tumor types. Moreover, in the subset of patients that relapsed to the brain in the first 5 years (*n *= 42), lower levels of *PTEN *expression were found to be associated with a shorter time to brain recurrence (1.6 versus 3.4 years; *P *= 0.003), even when adjusted for ER status (*P *= 0.001) and ER status plus subtype (*P *= 0.0003; Figure 8b). Finally, no association of *S6K *and *AKT-1, -2*, and -*3 *genes with outcome was observed (data not shown).

## Discussion

BCBMs represent one of the most challenging aspects in the clinical care of patients with advanced BC. Not only does intracranial recurrence limit survival, but associated symptoms also decrease functional status, limit independence, and negatively affect quality of life. No approved systemic therapies are available to treat patients with BCBMs, and it is unclear whether therapeutic targets, such as PI3K, differ between primary BC and BCBMs. In the present study, we explored the expression and prognostic implications of a panel of PI3K-pathway biomarkers, p-AKT, p-S6, and PTEN, in 52 BCBMs and 12 matched primary BCs. Our central goal was to improve our current understanding of the complex biology underlying BCBMs in hopes of guiding the future use of targeted agents to treat this aggressive disease. Our results show that the PI3K pathway is active in most BCBMs, regardless of IHC subtype; however, activation status does not appear to affect overall survival or survival after BCBMs in this cohort of patients. Interestingly, our secondary analyses indicate that the lack of PTEN expression may have prognostic value, independent of subtype. Moreover, among patients with aggressive TN BCBM, lack of PTEN expression may also be associated with worse overall survival.

Although alterations of the PI3K pathway are frequently observed in primary BC [25], different mechanisms of pathway activation exist [26]. Among them, activating PIK3CA mutations have been identified in about 15% to 30% of breast tumors and are more commonly associated with ER+ disease [27-31]. Conversely, alternative mechanisms of PI3K pathway activation, such as loss of PTEN and loss of the tumor-suppressor inositol polyphosphate 4-phosphatase type II (INPP4B), are more commonly associated with basal-like BC [27,32-37]. Although our data indicate that PI3K pathway activation in BCBM is not entirely subtype specific, lack of PTEN expression was more commonly observed in the TN and basal-like subtypes when compared with the other tumor types. Given that brain metastases across subtypes were included in this study, multiple mechanisms of PI3K activation (that is, PIK3CA mutations, PTEN loss, and/or INPP4B loss) may be responsible for the high levels of PI3K pathway activation observed in this cohort. Future studies aimed at identifying subtype-specific mechanisms of PI3K activation are certainly warranted, both in primary BC and BCBM, to refine our current understanding of the biologic processes driving this disease process.

The role of PI3K-pathway activation as a prognostic and/or predictive biomarker is under investigation. Although our primary analyses did not reveal associations between PI3K-pathway activation and overall survival or survival after BCBM, several secondary analyses are worthy of discussion. Our exploratory analysis indicates PTEN may be prognostic, with lack of PTEN expression being associated with more rapid time to disease recurrence (across subtypes) and worse overall survival in the TN subset of patients [38]. Interestingly, of the three biomarkers evaluated in this study, PTEN showed the highest concordance (83%) between matched primary BC and BCBM. In the clinical setting, biologic specimens from brain metastases are not commonly available, as resection is generally reserved for solitary lesions, and biopsies are reserved for cases with equivocal radiographic findings. Given the high concordance of PTEN status between primary BCs and their BCBMs, PTEN status in primary breast tumors may also be prognostic, and potentially predictive of distant and CNS recurrence. Confirming these findings in a large, unselected cohort of patients with primary breast tumor tissue available for PTEN testing would certainly be of value.

We recognize that the data presented in this study have several limitations. First, all patients included in this study underwent a neurosurgical procedure, so the population studied here might not be representative of all patients with BCBMs. Second, the sample size in this study is small, but comparable to previously reported studies evaluating BCBM tissues [39]. Although this is the largest study evaluating PI3K activation in BCBM tissues to date, subset analyses should be interpreted with caution because of the small sample size and multiple comparisons. A laudable future goal would be to validate these findings in a larger cohort; however, the inherent difficulty of obtaining brain metastasis tissues remains an obstacle. Thus, the development of clinically annotated brain metastases and primary BC tissue repositories housing both paraffin-embedded and fresh, frozen tissues should be a priority among the scientific community.

Finally, we used an IHC definition to identify the intrinsic molecular subtypes based on ER, PR, and HER2 status. We recognize that considerable discordance may exist between subtype assignment by IHC biomarkers and molecular profiling [40]. However, similar results were observed when we evaluated publicly available gene-expression data in more than 800 tumors in which molecular profiling had been performed [19,20]. Overall, this genomic analysis supports our IHC findings, in which *PTEN *expression was associated with time to distant and brain recurrence, basal-like tumors, and the development of BCBM.

## Conclusions

In summary, results of this study indicate that the PI3K pathway is active in the majority of BCBMs across the spectrum of IHC subtypes. Although expression of the PI3K pathway did not correlate with OS and survival after BCBM, loss of PTEN may hold prognostic and/or predictive value among this group of very high risk patients. Presently, small-molecule inhibitors of the PI3K pathway are in clinical development to treat multiple malignancies, including BC [41,42], and several cross the blood-brain barrier. Thus, inhibition of the PI3K pathway represents a promising therapeutic strategy for patients with BCBMs, with the ultimate goal of improving outcome and quality of life for patients diagnosed with this devastating disease.

## Abbreviations

95% CI: 95% confidence interval; AJCC: American Joint Committee on Cancer; ASCO/CAPS: American Society of Clinical Oncology ASCO)/College of American Pathologists; BCBM: breast cancer brain metastasis; CNS: central nervous system; CNS survival: CNS-specific survival; ER: estrogen receptor; FISH: fluorescence *in situ *hybridization; GSE: Gene Expression Omnibus Series; HER2: human epidermal growth factor receptor 2; HR: hormone receptor; HR: hazard ratio; IHC: immunohistochemistry; INPP4B: inositol polyphosphate-4-phosphatase; mTOR: mammalian target of rapamycin; OS: overall survival; p: phosphor; PIK3CA: phosphoinositide-3-kinase, catalytic, alpha polypeptide; PI3K: phosphatidylinositol 3-kinase; PR: progesterone receptor; PTEN: phosphatase and tensin homologue; SAS: Statistical Analysis Software; TNBC: triple-negative breast cancer; TTCNS: time to central nervous system recurrence; TTDR: time to distant recurrence; UNC: University of North Carolina at Chapel Hill; XRT: radiation.

## Competing interests

The authors declare that they have no competing interests.

## Authors' contributions

BA, KLB, CRM, and CKA designed the concept of the study; JG, KLB, CL, MGE, and CRM provided clinical data; EB, EH, KLB, and LAC provided tumor tissue samples; JCH provided gene-expression data; JG, CL, KF, and CRM performed pathologic examination and immunohistochemistry; BA, AMD, EB, EH, AP, JCH, and CKA performed data gathering and management; BA, AMD, AP, CRM, and CKA performed statistical analysis and interpretation; and BA, AMD, AP, CRM, and CKA wrote the manuscript. All authors reviewed the manuscript critically for important intellectual content and read and approved the final manuscript.

## References

[B1] Kirsch D Loeffler J Brain metastases in patients with breast cancer: new horizons Clin Breast Cancer 2005 6 115 124 10.3816/CBC.2005.n.013 16001989

[B2] Boogerd W Vos V Hart A Baris G Brain metastases in breast cancer; natural history, prognostic factors and outcome J Neurooncol 1993 15 165 174 10.1007/BF01053937 8509821

[B3] Lin N Bellon J Winer E CNS metastases in breast cancer J Clin Oncol 2004 22 3608 3617 10.1200/JCO.2004.01.175 15337811

[B4] Gasper L Recursive Partioning Analysis (RPA) of prognostic factors in three Radiation Oncology Treatment Group (RTOG) brain metastasis trials Int J Radiat Oncol Biol Phys 1997 37 745 751 10.1016/S0360-3016(96)00619-0 9128946

[B5] Engel J Determinants and prognosis of locoregional and distant progression in breast cancer Int J Radiat Oncol Biol Phys 2003 55 1186 1195 10.1016/S0360-3016(02)04476-0 12654426

[B6] Isakoff S Engelman J Irie H Luo J Brachmann S Pearline R Cantley L Brugge J Breast cancer-associated PI3KA mutations are oncogenic in mammary epithelial cells Cancer Res 2005 65 10992 11000 10.1158/0008-5472.CAN-05-2612 16322248

[B7] Bunney T Katan M Phosphoinositide signalling in cancer: beyond PI3K and PTEN Nat Rev Cancer 2010 10 342 352 10.1038/nrc2842 20414202

[B8] Dillon R White D Muller W The phosphatidyl inositol 3-kinase signaling network: implications for human breast cancer Oncogene 2007 26 1338 1345 10.1038/sj.onc.1210202 17322919

[B9] Levine D Bogomolniy F Yee C Lash A Barakat R Borgen P Boyd J Frequent mutation of the PI3KCA gene in ovarian and breast cancers Hum Cancer Biol 2005 11 2875 2878 10.1158/1078-0432.CCR-04-2142 15837735

[B10] Li S Rong M Grieu F Iacopetta B PI3KCA mutations in breast cancer are associated with poor outcome Breast Cancer Res Treat 2006 96 91 95 10.1007/s10549-005-9048-0 16317585

[B11] Tokunaga E Kimura Y Mashino K Oki E Kotaoka A Ohno S Morita M Kakeji Y Baba H Maehara Y Activation of PI3K/Akt signaling and hormone resistance in breast cancer Breast Cancer 2006 13 137 144 10.2325/jbcs.13.137 16755107

[B12] Vivanco I Sawyers C The phosphotidylinositol-3-kinase AKT pathway in human cancer Nat Rev Cancer 2002 2 489 501 10.1038/nrc839 12094235

[B13] Sansal I Sellers W The biology and clinical relevance of the PTEN tumor suppressor pathway J Clin Oncol 2004 22 2954 2963 10.1200/JCO.2004.02.141 15254063

[B14] Bose S Chandran S Mirocha J Bose N The AKT pathway in human breast cancer: a tissue-array-based analysis Modern Pathol 2006 19 238 245 10.1038/modpathol.3800525 16341149

[B15] Stemke-Hale K Gonzalez-Angulo AM Lluch A Neve RM Kuo WL Davies M Carey M Hu Z Guan Y Sahin A Symmans WF Pusztai L Nolden LK Horlings H Berns K Hung MC van de Vijver MJ Valero V Gray JW Bernards R Mills GB Hennessy BT An integrative genomic and proteomic analysis of PIK3CA, PTEN, and AKT mutations in breast cancer Cancer Res 2008 68 6084 6091 10.1158/0008-5472.CAN-07-6854 18676830 PMC2680495

[B16] Kesari S Ramakrishna N Sauvageot C Stiles C Wen P Targeted molecular therapy of malignant gliomas Curr Oncol Rep 2006 8 58 70 10.1007/s11912-006-0011-y 16464405

[B17] Mischel P Cloughesy T Targeted molecular therapy of GBM Brain Pathol 2003 13 52 61 12580545 10.1111/j.1750-3639.2003.tb00006.x PMC8095874

[B18] Andersen JN Sathyanarayanan S Di Bacco A Chi A Zhang T Chen AH Dolinski B Kraus M Roberts B Arthur W Klinghoffer RA Gargano D Li L Feldman I Lynch B Rush J Hendrickson RC Blume-Jensen P Paweletz CP Pathway-based identification of biomarkers for targeted therapeutics: personalized oncology with PI3K pathway inhibitors Sci Transl Med 2010 2 43 55 10.1126/scitranslmed.3001065 20686178

[B19] Harrell J Prat A Parker J Fan C He X Carey L Anders C Ewend M Perou C Genomic analysis identifies unique signatures predictive of brain, lung, and liver relapse Breast Cancer Res Treat 2011 in press 10.1007/s10549-011-1619-7 PMC3303043 21671017

[B20] Zhang XHF Wang Q Gerald W Hudis CA Norton L Smid M Foekens JA Massagué J Latent bone metastasis in breast cancer tied to Src-dependent survival signals Cancer Cell 2009 16 67 78 10.1016/j.ccr.2009.05.017 19573813 PMC2749247

[B21] Anders CK Deal AM Khorram C Meng H Burrows E Livasy C Fritchie K Ewend MG Perou CM Carey LA The prognostic contribution of intrinsic breast cancer subtype, race, and age among patients with brain metastases J Clin Oncol 2010 28 abstr 1027 10.1002/cncr.25746 PMC4265570 21472708

[B22] Nam B-H Kim SY Han H-S Kwon Y Lee KS Kim TH Ro J Breast cancer subtypes and survival in patients with brain metastases Breast Cancer Res 2008 10 1 8 10.1186/bcr1870 PMC2374976 18307763

[B23] Niwinska A Murawska M Pogoda K Breast cancer brain metastases: differences in survival depending on biological subtype, RPA RTOG prognostic class and systemic treatment after whole-brain radiotherapy (WBRT) Ann Oncol 2010 21 942 948 Epub 2009 Oct 19 10.1093/annonc/mdp407 19840953

[B24] Gustin JP Karakas B Weiss MB Abukhdeir AM Lauring J Garay JP Cosgrove D Tamaki A Konishi H Konishi Y Mohseni M Wang G Rosen DM Denmeade SR Higgins MJ Vitolo MI Bachman KE Park BH Knockin of mutant PIK3CA activates multiple oncogenic pathways Proc Natl Acad Sci USA 2009 106 2835 2840 10.1073/pnas.0813351106 19196980 PMC2636736

[B25] Engelman J Targeting PI3K signalling in cancer: opportunities, challenges and limitations Nat Rev Cancer 2009 9 550 562 10.1038/nrc2664 19629070

[B26] Bader A Kang S Zhao L Vogt P Oncogenic PI3K deregulates transcription and translation Nat Rev Cancer 2005 5 921 929 10.1038/nrc1753 16341083

[B27] Kalinsky K Jacks LM Heguy A Patil S Drobnjak M Bhanot UK Hedvat CV Traina TA Solit D Gerald W Moynahan ME PIK3CA mutation associates with improved outcome in breast cancer Clin Cancer Res 2009 15 5049 5059 10.1158/1078-0432.CCR-09-0632 19671852

[B28] Liedtke C Cardone L Tordai A Yan K Gomez HL Figureoa LJ Hubbard RE Valero V Souchon EA Symmans WF Hortobagyi GN Bardelli A Pusztai L PIK3CA-activating mutations and chemotherapy sensitivity in stage II-III breast cancer Breast Cancer Res 2008 10 R27 10.1186/bcr1984 18371219 PMC2397526

[B29] Brugge J Hung M Mills G A new mutational aktivation in the PI3K pathway Cancer Cell 2007 12 104 107 10.1016/j.ccr.2007.07.014 17692802

[B30] Saal LH Holm K Maurer M Memeo L Su T Wang X Yu JS Malmström PO Mansukhani M Enoksson J Hibshoosh H Borg A Parsons R PI3KCA mutations correlate with hormone receptors, node metastasis, and ERBB2, and are mututally exclusive with PTEN loss in human breast carcinoma Cancer Res 2005 65 2554 2559 10.1158/0008-5472-CAN-04-3913 15805248

[B31] Samuels Y Wang Z Bardelli A Silliman N Ptak J Szabo S High frequency mutations of the PI3KCA gene in human cancers Science 2004 304 554 10.1126/science.1096502 15016963

[B32] Aleskandarany M Rakha E Ahmed M Powe D Ellis I Green A Clinicopathologic and molecular significance of phospho-Akt expression in early invasive breast cancer Breast Cancer Res Treat 2010 10.1007/s10549-010-1012-y 20617378

[B33] Aleskandarany M Rakha E Ahmed M Powe D Paish E Macmillan R Ellis I Green A PIK3CA expression in invasive breast cancer: a biomarker of poor prognosis Breast Cancer Res Treat 2010 122 45 53 10.1007/s10549-009-0508-9 19701705

[B34] Akcakanat A Sahin A Shaye A Velasco M Meric-Bernstam F Comparison of Akt/mTOR signaling in primary breast tumors and matched distant metastases Cancer 2008 112 2352 2358 10.1002/cncr.23456 18386830 PMC2819051

[B35] Capodanno A Camerini A Orlandini C Baldini E Resta M Bevilacqua G Collecchi P Dysregulated PI3K/Akt/PTEN pathway is a marker of a short disease-free survival in node-negative breast carcinoma Hum Pathol 2009 40 1408 1417 10.1016/j.humpath.2009.02.005 19428048

[B36] Fedele CG Ooms LM Ho M Vieusseux J O'Toole SA Millar EK Lopez-Knowles E Sriratana A Gurung R Baglietto L Giles GG Bailey CG Rasko JE Shields BJ Price JT Majerus PW Sutherland RL Tiganis T McLean CA Mitchell CA Inositol polyphosphate 4-phosphatase II regulates PI3K/Akt signaling and is lost in human basal-like breast cancers Proc Natl Acad Sci USA 2010 107 22231 22236 10.1073/pnas.1015245107 21127264 PMC3009830

[B37] Gewinner C Wang ZC Richardson A Teruya-Feldstein J Etemadmoghadam D Bowtell D Barretina J Lin WM Rameh L Salmena L Pandolfi PP Cantley LC Evidence that inositol polyphosphate 4-phosphatase type II is a tumor suppressor that inhibits PI3K signaling Cancer Cell 2009 16 115 125 10.1016/j.ccr.2009.06.006 19647222 PMC2957372

[B38] Pérez-Tenorio G Alkhori L Olsson B Waltersson M Nordenskjöld B Rutqvist L Skoog L Stål O PIK3CA mutations and PTEN loss correlate with similar prognostic factors and are not mutually exclusive in breast cancer Clin Cancer Res 2007 13 3577 3584 10.1158/1078-0432.CCR-06-1609 17575221

[B39] Brogi E Murray M Nehhozina T Akram M Cranor M Seidman A Clinical and pathologic characteristics of brain metastases of breast carcinoma: a study of 74 patients San Antonio Breast Cancer Symposium 2006 Abstract 4003

[B40] Prat A Perou CM Deconstructing the molecular portraits of breast cancer Mol Oncol 2011 5 5 23 10.1016/j.molonc.2010.11.003 21147047 PMC5528267

[B41] Garcia-Echeverria C Sellers W Drug discovery approaches targeting the PI3K/Akt pathway in cancer Oncogene 2008 27 5511 5526 10.1038/onc.2008.246 18794885

[B42] Baselga J Targeting the phosphoinositide-3 (PI3) kinase pathway in breast cancer Oncologist 2011 16 suppl 1 12 9 21278436 10.1634/theoncologist.2011-S1-12

